# Clinical judgment of GPs for the diagnosis of dementia: a diagnostic test accuracy study

**DOI:** 10.3399/BJGPO.2021.0058

**Published:** 2021-09-15

**Authors:** Samuel Thomas Creavin, Judy Haworth, Mark Fish, Sarah Cullum, Anthony Bayer, Sarah Purdy, Yoav Ben-Shlomo

**Affiliations:** 1 Population Health Sciences, University of Bristol, Bristol, UK; 2 Royal Devon and Exeter NHS Foundation Trust, Exeter, UK; 3 Depatment of Psychological Medicine, School of Medicine, The University of Auckland, Grafton, New Zealand; 4 School of Medicine, Cardiff University, Cardiff, UK

**Keywords:** dementia, general practice, sensitivity and specificity, medical history taking, symptom assessment

## Abstract

**Background:**

GPs often report using clinical judgment to diagnose dementia.

**Aim:**

To investigate the accuracy of GPs’ clinical judgment for the diagnosis of dementia.

**Design & setting:**

Diagnostic test accuracy study, recruiting from 21 practices around Bristol, UK.

**Method:**

The clinical judgment of the treating GP (index test) was based on the information immediately available at their initial consultation with a person aged ≥70 years who had cognitive symptoms. The reference standard was an assessment by a specialist clinician, based on a standardised clinical examination and made according to the 10th revision of the International Classification of Diseases (ICD-10) criteria for dementia.

**Results:**

A total of 240 people were recruited, with a median age of 80 years (interquartile range [IQR] 75–84 years), of whom 126 (53%) were men and 132 (55%) had dementia. The median duration of symptoms was 24 months (IQR 12–36 months) and the median Addenbrooke's Cognitive Examination III (ACE-III) score was 75 (IQR 65–87). GP clinical judgment had sensitivity 56% (95% confidence interval [CI] = 47% to 65%) and specificity 89% (95% CI = 81% to 94%). Positive likelihood ratio was higher in people aged 70–79 years (6.5, 95% CI = 2.9 to 15) compared with people aged ≥80 years (3.6, 95% CI = 1.7 to 7.6), and in women (10.4, 95% CI = 3.4 to 31.7) compared with men (3.2, 95% CI = 1.7 to 6.2), whereas the negative likelihood ratio was similar in all groups.

**Conclusion:**

A GP clinical judgment of dementia is specific, but confirmatory testing is needed to exclude dementia in symptomatic people whom GPs judge as not having dementia.

## How this fits in

Previous studies in this area have investigated the accuracy of GP clinical judgment as a screening test for dementia in unselected people attending a primary care clinic or as a retrospective test based on their knowledge of their patient. Some studies have derived the accuracy of judgment from the medical records, which may not reflect the judgment of the clinician. The role of the GP in supporting a more effective route to diagnosis for people with dementia is a research priority for patients, carers, and clinicians. This study shows that, in a symptomatic older adult, clinical judgment may be useful for helping to confirm a diagnosis of dementia, but GP judgment should not by itself be used to exclude dementia.

## Introduction

The James Lind Alliance has identified the role of general practice in supporting a more effective route to diagnosis of dementia as a priority for health research.^1^ People with symptoms of dementia have historically faced long delays to get an assessment and an explanation for their symptoms.^2^ Approaches to address waiting lists have included psychiatrists supporting primary care memory clinics,^3^ integrated one-stop clinics,^4^ and training GPs to make a diagnosis in uncomplicated cases,^5,6^ which is supported by the National Institute for Health and Care Excellence (NICE).^7^ Some GPs have in the past been hesitant about diagnosing dementia when there is no disease-modifying treatment,^8^ and disclosure of a diagnosis can still be problematic, especially if the affected person is not seeking help.^9^ The situation has been complicated in the UK by controversial policies that have funded case-finding for dementia.^10–12^ Formally evaluating cognition takes time and familiarity with tests. A GP could use a range of brief cognitive assessments^13^ to evaluate a person with symptoms of dementia, and national guidelines differ on which test to use.^14,15^ Instead, GPs report using non-standardised processes^16^ such as clinical judgment^17^ to diagnose dementia. The sensitivity of GP clinical judgment for diagnosing dementia has been reported as between 51%^18^ and 100%,^19^ and the specificity ranges from 58%^20^ to 100%.^19^

Previous studies to investigate the accuracy of GP clinical judgment have typically suffered from one of two significant limitations.^21^ First, a definition of clinical judgment that is of unclear relevance to practice, such as judgment in hindsight, or documentation of recorded diagnoses in the medical record that are systematically incomplete.^22^ Second, sampling unselected people attending general practice regardless of symptoms, which is more akin to screening. The aim of this study was to address these limitations of earlier studies and investigate the prospective diagnostic accuracy of GP clinical judgment for the diagnosis of dementia syndrome in symptomatic people aged >70 years.^23^

## Method

### Population

Participants were recruited from 21 participating GP surgeries in the Bristol, North Somerset, and South Gloucestershire (BNSSG) area, which is a diverse geographic area within 15 miles of the city of Bristol, covering a total population of around 900 000 people across 82 GP practices. Research clinics were in four participating GP surgeries, strategically located for accessibility. It was calculated that a minimum sample size of 200 was needed, based on a specificity of 95% in prior studies, and a 75% prevalence of dementia in local memory clinic data.^24^

### Inclusion and exclusion criteria

Participants were people with cognitive symptoms but no prior diagnosis of dementia, aged >70 years, and who had been referred by their GP to this research study. Cognitive symptoms were not specified but generally include disturbance in memory, language, executive function, behaviour, and visuospatial skills.^25^ Symptoms were required to be present for at least 6 months, and could be reported by the person themselves, a family member, a professional, or another person. There was no severity threshold. Cognitive problems did not need to be the focus of the consultation and (as routine practice) GPs could enquire about cognition if they perceived a problem. Symptom duration was determined from the clinical history. An accompanying informant was mandatory. All participants were offered free accessible transport and translation services. People were excluded if they had a known neurological disorder (that is, Parkinsonism, multiple sclerosis, learning disability, Huntington’s disease), were registered as blind, had profound deafness (that is, were unable to use a telephone), had a psychiatric disorder requiring current secondary care input, or if cognitive symptoms were either rapidly progressive or coincident with neurological disturbance. People with cognitive problems that were so advanced that they were unable to consent were excluded, as they were judged by a lay advisory group to find the research process overly burdensome. GPs were encouraged to make a clinical judgment and refer a consecutive series of all eligible patients with cognitive symptoms to the study, regardless of what their clinical judgment was or of any test results. GPs gave study information including a leaflet, and obtained verbal consent to share contact details with the study on a referral form, including the person's age, sex, contact details, and the GP's clinical judgment. The study team contacted people referred by GPs to re-confirm eligibility, provide further written study details, and offer a research clinic appointment. The research team took written consent from all participants.

### Index test of clinical judgment

The referring GP recorded their clinical judgment using an electronic referral form during a consultation with their patient about cognitive symptoms. Clinical judgment was operationalised as 'normal' cognition, 'cognitive impairment not dementia (CIND)', or 'dementia' as options for response to the question 'Is your gut feeling that this person has ___ ?'. GPs were not specially trained, were not required to arrange any test, and could refer people simultaneously or subsequently to NHS services. The study team contacted the practice at least three times to obtain any missing referral data.

### Reference standard

At the research clinic, a single specialist physician conducted a standardised assessment lasting approximately 60 minutes, comprising clinical history, the ACE-III,^26^ Brief Assessment Schedule Depression Cards (BASDEC),^27^ and the informant-completed Bristol Activities of Daily Living (BADL) Questionnaire.^28^ The specialist was not aware of other test results, including GP judgment or investigations. The reference standard was based on the evaluation of the specialist physician for dementia, according to ICD-10 criteria^29^ for each individual patient. Specific cut-offs on the aforementioned measures were not used and the expert used their integrated assessment to reach a diagnosis. CIND was diagnosed by the same expert and included Petersen mild cognitive impairment (MCI)^30^ and other causes of cognitive impairment that met neither criteria for ICD-10 dementia nor Petersen MCI, such as traumatic brain injury or affective disorder. Medical records were reviewed for all participants 6 months after the research clinic to identify any subsequent information that would contradict this judgment. A second specialist adjudicated cases where there was diagnostic uncertainty at the research clinic using the initial specialist assessment and the medical record review; the second specialist also did not have access to the GP judgment. Study data were electronically entered and managed using REDCap (Research Electronic Data Capture) hosted at the University of Bristol.^31^

### Statistical methods

Separate logistic regression analyses were used with non-participation (referred by GP but not taking part) as the dependent variable and GP judgment, age (in years), and female sex as the independent variables to test the hypothesis of no association with these variables. Time from referral to appointment was described using median and IQR, and logistic regression was used to test the hypothesis of no association between time to appointment (in days) and dementia (as the dependent variable). Measures of diagnostic test accuracy were calculated together with 95% CIs, for GP judgment of dementia against reference standard of dementia. Sensitivity analyses were done to explore whether accuracy varied by age (<80 years and ≥80 years, since prediction models perform differently in these age groups)^32^ and sex. Cochran’s Q test was used to test the hypothesis of no difference in likelihood ratios between groups.^33^ This diagnostic test accuracy study is reported in line with STARDdem guidelines.^34^

## Results

### Participants

**Figure 1.**
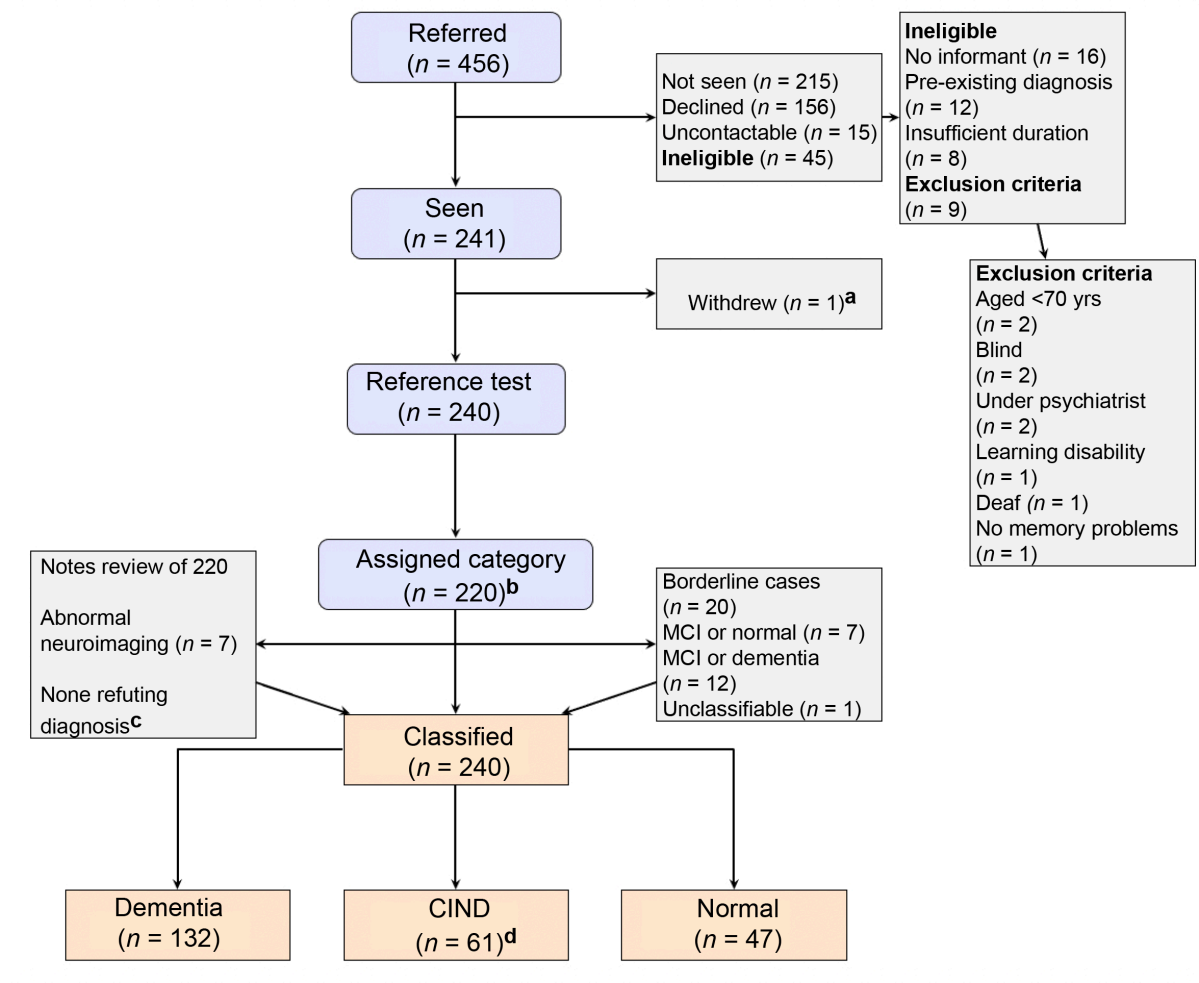
STARDdem flowchart for inclusion of participants in the study. CIND = cognitive impairment not dementia. ICD-10 = International Classification of Diseases, 10th revision. MCI = mild cognitive impairment. ^a^One person had to withdraw part way through the reference test as they were acutely ill. ^b^Dementia according to ICD-10.^29^ ^c^One person met criteria for ICD-10 dementia and also had features of normal pressure hydrocephalus. Expert review endorsed a reference standard diagnosis of dementia. ^d^Of 61 with CIND: 59 met criteria for Peterson MCI;^30^ 1 affective disorder; 1 brain injury.

Recruitment took place between March 2015 and May 2017. Figure 1 shows a flowchart for inclusion in the study. The theoretically 'eligible' figure of 1735 people was derived from the age-specific incidence of dementia^35^ and the demographics of the population in the participating practices (34 956 people aged >70 years).^36^ The number approached is unknown. One person who consented withdrew before any data were collected because they were acutely ill. Of the 240 with available data, there were 20 borderline cases that were adjudicated by a second specialist. The 240 people were classified by the reference standard as: 'normal' cognition (*n* = 47); 'dementia' (*n* = 132, of whom one had DSM-5 but not ICD-10 because they had subjective but not objective amnesia); or 'CIND' (*n* = 61), of whom 59 met criteria for MCI (one affective disorder, one brain injury). Compared with people who participated, there was little evidence of an association between non-participation and a GP clinical judgment of CIND (odds ratio [OR] 1.2; 95% CI = 0.55 to 2.41) or dementia (OR 1.9; 95% CI = 0.90 to 3.93). Compared with people who participated, non-participants were older (OR per year 1.08; 95% CI = 1.04 to 1.12), or more often female (OR 1.88; 95% CI = 1.21 to 2.92). The median time between referral (clinical judgment) and the clinic appointment (reference standard) was 47 days (IQR 30–72 days). The longest interval was 177 days, owing to difficulties attending earlier appointments. There was no association between time from referral to appointment and dementia (OR per day 1.0; 95% CI = 0.99 to 1.01). Table 1 shows the demographics of participants and shows a cross-tabulation of GP opinion against the reference standard, allowing derivation of diagnostic accuracy of clinical judgment for both CIND and dementia.

**Table 1.** Characteristics of participants by cognitive category

Characteristic	Cognitive category^a^
Dementia	CIND	Normal cognition
*n* = 132	*n* = 61	*n* = 47
Sex, *n* (column %)			
Male	68 (52)	35 (57)	23 (49)
Female	64 (49)	26 (43)	24 (51)
Median age, years (IQR)			
At clinic	82 (77–86)	80 (75–83)	75 (72–80)
Left school	15 (15–16)	15 (15–16)	16 (15–16)
Retired	60 (58–65)	60 (58–67)	61 (58–65)
Median symptom onset, months (IQR)			
Time ago	24 (12–36)	18 (12–24)	21 (12–36)
Type, *n* (column %)			
Gradual	111 (84)	55 (90)	43 (91)
Sudden	13 (10)	5 (8)	0 (-)
Uncertain	8 (6)	1 (1)	4 (9)
Course, *n* (column %			
Progressive	111 (84)	42 (69)	29 (62)
Stepwise	2 (2)	0 (-)	0 (-)
Regressive	1 (1)	1 (2)	1 (2)
Static	5 (4)	7 (11)	9 (19)
Uncertain	13 (10)	11 (18)	8 (17)
Fluctuation, *n* (column %			
None	112 (85)	53 (87)	45 (96)
Within 1 day	12 (9)	5 (8)	1 (2)
Over several days	8 (6)	3 (5)	1 (2)
Median ACE-III score (IQR)			
Total (max 100)	69 (61–74)	82 (76–87)	93 (90–95)
GP opinion, *n* (row %)			
Normal cognition	6 (18)	9 (26)	19 (56)
CIND	52 (43)	41 (34)	27 (23)
Dementia	74 (86)	11 (13)	1 (1)

^a^Dementia according to International Classification of Diseases 10th revision (ICD-10) definition; mild cognitive impairment (MCI) according to Petersen definition.

ACE-III = Addenbrookes’ Cognitive Examination III. CIND = Cognitive impairment not dementia.

Two people could not complete the ACE-III because English was not their first language; they had both declined an interpreter. In both cases sufficient information was available from other parts of the assessment for a categorisation about cognition to be made (one had normal cognition, one had dementia). For the 238 people who had an ACE-III score, the median was 75 (interquartile range 65–87). Referring GPs judged that 34 people had normal cognition, 86 had dementia, and 120 had CIND; the one person who withdrew from the study, owing to acute illness, was judged by the referring GP to have CIND. People whom GPs judged as having dementia had a total ACE-III score IQR of 60–74, with a 90th centile of 81/100 and highest score of 95/100. Similarly, people whom GPs judged as having CIND had an ACE-III score IQR 71–89.

### Diagnostic accuracy

Table 2 shows the diagnostic accuracy for GP judgment for dementia. The sensitivity of GP judgment was 56% (95% CI = 47% to 65%) and the specificity was 89% (95% CI = 81% to 94%). Clinical judgment was more useful for ruling in dementia, than ruling it out, with higher specificity and positive predictive value than sensitivity and negative predictive value. In people aged ≥80 years, clinical judgment had similar sensitivity (*P* = 0.296) and specificity (*P* = 0.798) to those aged <80 years. There was weak evidence that clinical judgment in women had a higher specificity (*P* = 0.074) and a higher sensitivity (*P* = 0.064) than clinical judgment in men.

**Table 2.** Accuracy of GP judgment for the diagnosis of dementia

	Sensitivity	Specificity	PPV	NPV	LRP	LRN
(95% CI)	(95% CI)	(95% CI)	(95% CI)	(95% CI)	(95% CI)
GP judgment(*n*= 240)	56 (47 to 65)	89 (81 to 94)	86 (77 to 93)	62 (54 to 70)	5.1 (2.9 to 8.8)	0.49 (0.40 to 0.61)
Age ≥80 years(*n*= 123)	57 (45 to 67)	84 (67 to 94)	89 (77 to 96)	46 (34 to 59)	3.6 (1.7 to 7.6)	0.52 (0.39 to 0.68)
Age <80 years(*n*= 117)	55 (40 to 70)	91 (82 to 97)	81 (64 to 93)	75 (65 to 84)	6.5 (2.9 to 15)	0.49 (0.35 to 0.68)
Men(*n*= 126)	50 (38 to 62)	85 (73 to 93)	79 (64 to 90)	59 (48 to 70)	3.2 (1.7 to 6.2)	0.59 (0.46 to 0.77)
Women(*n*= 114)	63 (50 to 74)	94 (84 to 99)	93 (81 to 99)	66 (54 to 77)	10.4 (3.4 to 31.7)	0.40 (0.29 to 0.55)

LRP = positive likelihood ratio. LRN = negative likelihood ratio. NPV = negative predictive value. PPV = positive predictive value.

## Discussion

### Summary

From 21 participating GP surgeries, 456 people were referred and 240 were evaluated. Of these, 132 (55%; 95% CI = 48% to 61%) had dementia. Clinical judgment as a single test had a positive likelihood ratio (LRP) of 5 (95% CI = 3 to 9) and a negative likelihood ratio (LRN) of 0.5 (95% CI = 0.4 to 0.6) for the target condition dementia. People whom GPs judged as having dementia had a total ACE-III score IQR of 60–74, and those whom they judged as having MCI had a total ACE-III IQR 71–89. This compares with published ACE-III thresholds of <82 for dementia^37^ and <88 for MCI,^37^ and suggests that in this study, GPs are not being overly restrictive in their judgment for dementia, or liberal in their judgment for CIND.

### Strengths and limitations

The patient selection in the current study closely reflects real-world clinical practice in the UK, with efforts to avoid exclusion based on language, transport, or appointment availability. Participants were included with a range of GP opinions about the presence of cognitive impairment in people who had presented with symptoms in a consultation; typically 2.5 problems are discussed per appointment.^38^ The index test reflects an average measure of diagnostic accuracy for an estimated 142 whole-time equivalent GPs working in different settings,^39^ who were not specially trained. GPs were instructed not to use any formal test to inform their judgment, but it is possible that brief cognitive tests, such as the General Practitioner Assessment of Cognition (GPCOG),^40^ may have been occasionally used. Based on previous studies, clinical judgment is likely to be based on rules of thumb,^16^ not formal tests,^17^ and information on referral forms indicated that judgment was informed by 'face-to-face presentation'. The interval between clinical judgment and the reference standard was unlikely to be associated with a significant progression in cognitive impairment.^15^ The index test for all consenting participants was fully verified, follow-up data were obtained after 6 months, and uncertain cases were adjudicated.

There was no evidence of selective participation by cognitive status, but non-participants may differ in other unmeasured ways that affect diagnostic accuracy. As reported in the Results, it is estimated that up to 1735 people in the study population would have developed symptoms in the study period, but it is unknown how many of these would have presented to their GP. The authors have no data on recruitment bias, but dementia was less prevalent than they predicted based on local memory clinic data, suggesting a lower threshold for referral to the study. Any systematic selection bias in who GPs referred to the study (such as excluding more frail people) would limit the generalisability of the findings to that group. An important limitation is that despite providing translation services, the population was largely White, native English-speakers. In addition, the CIs for the subgroups are still wide. People with advanced cognitive impairment who could not consent were excluded, so the findings cannot be generalised to that group, although it is likely that GPs would be more sensitive in identifying cognitive impairment at a more advanced stage.

### Comparison with existing literature

Table 3 summarises the features of this study compared with the existing literature.^41,42^ A major strength of this study for applicability to practice is that it is one of only two studies to evaluate symptomatic people. The present study has the smallest number undergoing the index test, but only one other study has complete verification by the reference standard.^43^ The present study has lower sensitivity and higher specificity than the French study,^20^ but this could be because the French study verified only 26% of people who underwent the index test (where participating GPs referred five patients per GP over 2 years), or because other studies did not require participants to be symptomatic and consequently had a lower prevalence of dementia (ranging 2%–29%).^44–47^

**Table 3.** Summary of seven studies investigating GP judgment for the diagnosis of dementia

	Mannheim^44^	Sydney^45^	Hawaii^43^	Antwerp^46^	AgeCoDe^47^	France^20^	This study
**Participant** **selection**							
Series	C	C	C	C	R	C	C
Symptomatic	No	No^a^	No	No	No	Yes^a^	Yes
**Characteristics of participants**							
Number (index test)	3721	433	303	1003	3242	1453	240
Mean age (years)	76	85	75	75	80	81	80
% Female	70	84	63	63	66	71	47
% with dementia	29	25	9	2	2	50	55
**Target condition and verification with reference standard**							
Verified *n*	407	105	303	101	22	385	240
Verified %	11	24	100	1	70	26	100
**GP judgment (%)**							
Not impaired	36	76	33	–	94	48	14
Cognitive impairment	41	–	–	–	–	–	40
Dementia	23	19	33	–	6	26	36
Uncertain	–	5	33	–	–	22	–
**Diagnostic accuracy of clinical judgment for dementia**							
Sensitivity	91	42	–	100	51	73	56
Specificity	76	89	–	100	96	58	89

C = consecutive. R = random; symptomatic: symptoms required for participation. % verified = number underwent reference test or number underwent index test. % with dementia = number with dementia or number verified.

a Participants were not presenting with symptoms but GPs were asked to maximise the inclusion of people with suspected dementia that was not reported.

### Implications for practice

The accuracy of clinical judgment was comparable to other brief cognitive tests, many of which are now subject to licensing restrictions. The test characteristics of clinical judgment would support an approach to subsequent testing; for example, where highly sensitive tests are performed in people whom GPs judge as not having dementia, but there is significant patient concern (to rule out disease); and where very highly specific, but minimally burdensome tests are done in people whom GPs do think have dementia. This would be a change to current practice where cognitive testing is typically done with the same tests regardless of GP judgment.
